# Phylogenetic analysis and protein structure modelling identifies distinct Ca^2+^/Cation antiporters and conservation of gene family structure within Arabidopsis and rice species

**DOI:** 10.1186/s12284-016-0075-8

**Published:** 2016-02-01

**Authors:** Jon K. Pittman, Kendal D. Hirschi

**Affiliations:** Faculty of Life Sciences, The University of Manchester, Michael Smith Building, Oxford Road, Manchester, M13 9PT UK; USDA/ARS Children’s Nutrition Research Center, Baylor College of Medicine, 1100 Bates Street, Houston, TX 77030 USA

**Keywords:** Cation transport, Proton/calcium exchanger, Sodium/calcium exchanger, Magnesium/proton exchanger, Phylogenetics, Protein structure

## Abstract

**Background:**

The Ca^2+^/Cation Antiporter (CaCA) superfamily is an ancient and widespread family of ion-coupled cation transporters found in nearly all kingdoms of life. In animals, K^+^-dependent and K^+^-indendent Na^+^/Ca^2+^ exchangers (NCKX and NCX) are important CaCA members. Recently it was proposed that all rice and Arabidopsis CaCA proteins should be classified as NCX proteins. Here we performed phylogenetic analysis of CaCA genes and protein structure homology modelling to further characterise members of this transporter superfamily.

**Findings:**

Phylogenetic analysis of rice and Arabidopsis CaCAs in comparison with selected CaCA members from non-plant species demonstrated that these genes form clearly distinct families, with the H^+^/Cation exchanger (CAX) and cation/Ca^2+^ exchanger (CCX) families dominant in higher plants but the NCKX and NCX families absent. NCX-related Mg^2+^/H^+^ exchanger (MHX) and CAX-related Na^+^/Ca^2+^ exchanger-like (NCL) proteins are instead present. Analysis of genomes of ten closely-related rice species and four Arabidopsis-related species found that CaCA gene family structures are highly conserved within related plants, apart from minor variation. Protein structures were modelled for OsCAX1a and OsMHX1. Despite exhibiting broad structural conservation, there are clear structural differences observed between the different CaCA types.

**Conclusions:**

Members of the CaCA superfamily form clearly distinct families with different phylogenetic, structural and functional characteristics, and therefore should not be simply classified as NCX proteins, which should remain as a separate gene family.

**Electronic supplementary material:**

The online version of this article (doi:10.1186/s12284-016-0075-8) contains supplementary material, which is available to authorized users.

## Findings

### The Ca^2+^/Cation antiporter (CaCA) superfamily

Control of ion concentrations is critical to cellular function. Such ion homeostasis is dependent on transporters, including ion-coupled transporters like the Na^+^/Ca^2+^ exchanger (NCX), Na^+^/Ca^2+^, K^+^ exchanger (NCKX), cation/Ca^2+^ exchanger (CCX) and H^+^/Cation exchanger (CAX), which are members of the CaCA superfamily (Cai and Lytton 2004). CaCAs are abundant throughout life; CAX and the prokaryotic-specific YRBG-type exchangers are abundant in bacteria, while NCX, NCKX and CCX genes are abundant in animals (Cai and Lytton 2004; Cai and Clapham 2012; Emery et al. 2012). In the land plant lineage NCKX and NCX have been lost but other types, including CAX, CCX and MHX (Mg^2+^/H^+^ exchanger) are present within all plants (Shigaki et al. 2006; Emery et al. 2012; Gaash et al. 2013). Many plant CaCAs have been functionally characterised and have key roles in cation transport and homeostasis (Hirschi et al. 1996; Shaul et al. 1999; Hirschi et al. 2000; Kamiya et al. 2005; Morris et al. 2008; Wang et al. 2012). Recently, Singh et al. (2015) performed phylogenetic analysis of CaCA genes from rice and Arabidopsis, and argued that all genes should be classified as members of the NCX family due to the ubiquitous presence of the so-called ‘NCX domain’ and tight phylogenetic relationship. Here we have performed further phylogenetic analysis of rice and Arabidopsis CaCAs, along with protein structure modelling to demonstrate that CaCA members form clearly distinct families with different structural and functional characteristics, and are not all NCXs. In addition, we have examined the genomes of closely related rice and Arabidopsis species to identify the conservation of CaCA genes within these genera.

### Phylogenetic analysis supports distinct CaCA gene families

Clear separation of CaCA family members within a phylogenetic tree is more apparent when members from each family are included, including NCKX and YRBG genes absent in higher plants. Therefore a phylogenetic tree was generated for rice (*Oryza sativa* Japonica) and Arabidopsis (*Arabidopsis thaliana*) CaCAs and with members from chosen species throughout life (see Additional file 1: Methods; Additional file 2: Table S1).

**Fig. 1:**
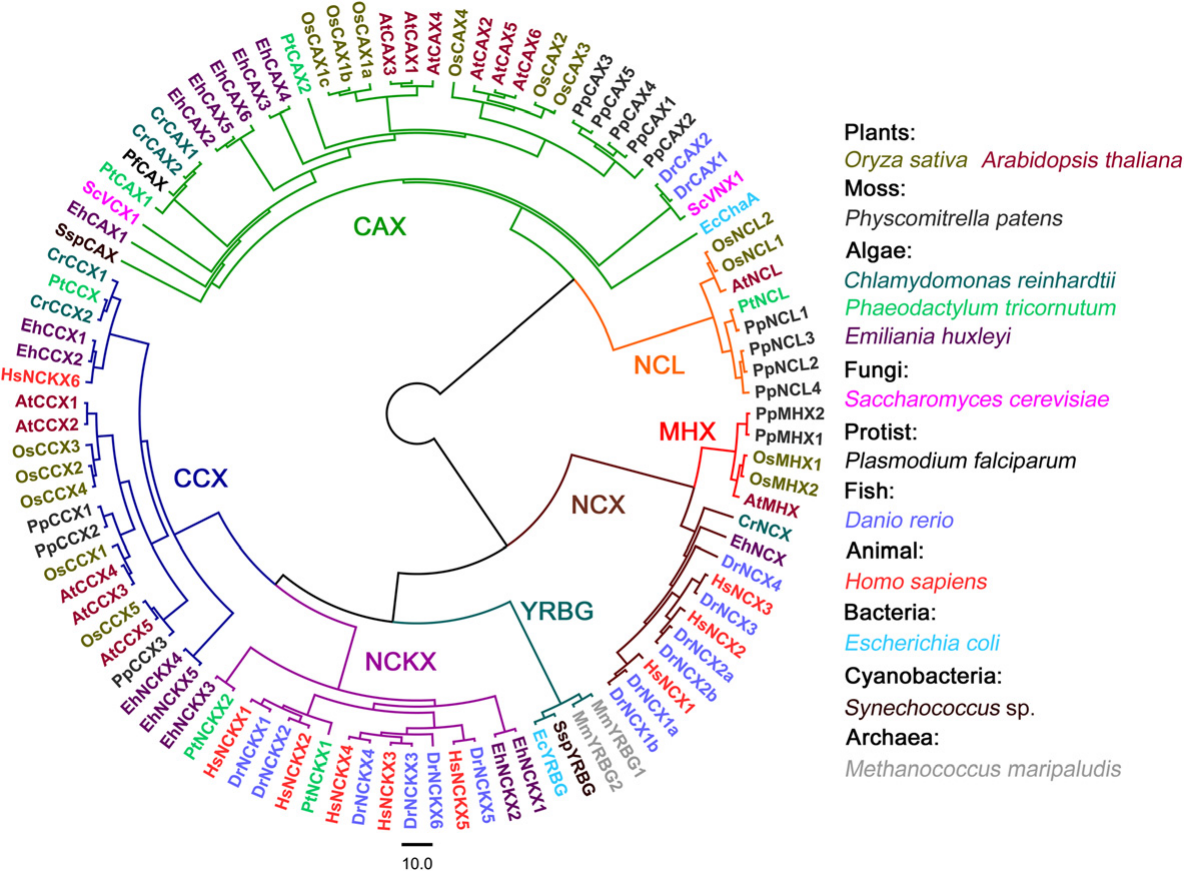
Phylogenetic analysis of the CaCA superfamily. The tree was derived from alignments of conserved ‘core domain’ hydrophobic region amino acid sequences obtained from the genomes of rice and Arabidopsis in comparison with selected algae, fungal, protist, animal, bacterial and archaebacterial species, and constructed using the maximum likelihood method. A consensus tree following 1000 bootstrap replications are shown. Bootstrap percentage values are shown in Additional file 4: Figure S2. The branch length scale bar indicates the evolutionary distance of ten amino acid substitutions per site

CaCA proteins have a conserved core structure of ten transmembrane (TM) helices and two conserved α-repeat regions within TM 2–3 and 7–8, which include residues for cation binding (Additional file 3: Figure S1a). High variability in amino acid sequence between distinct CaCAs, particularly within tail regions, means that full length sequences generate poor alignments and potentially inaccurate phylogenetic reconstruction. Therefore three sets of trees were compared: those generated from full-length sequence; ‘core domain’ sequence lacking tail sequence before TM1 and after TM10; and α2-repeat region sequence, which is conserved in all sequences, including NCL (Na^+^/Ca^2+^ exchanger-like) sequences, which lack the α1-repeat (Emery et al. 2012; Wang et al. 2012). All trees showed clear separation between the four major CaCA families: CAX, NCX, NCKX and CCX (Additional file 4: Figure S2), but YRBG genes were only clearly separated in the core domain tree (Fig. 1). MHX genes, which are unique to land plants and present instead of animal-like NCXs (Emery et al. 2012; Gaash et al. 2013), are shown as a separate clade within the NCX family cluster. NCL genes have been identified as new CaCA members very recently (Wang et al. 2012) and appear to be found only in photosynthetic organisms (Emery et al. 2012). In full length and core domain trees, NCLs arguably form a distinct family (Additional file 4: Figure S2), but in the α2-repeat tree, NCL genes were tightly grouped with CAXs, agreeing with previous analysis that despite exhibiting Na^+^/Ca^2+^ exchange activity (Li et al. 2016), AtNCL and related NCLs are more closely related to CAXs than NCXs (Emery et al. 2012).

**Fig. 2:**
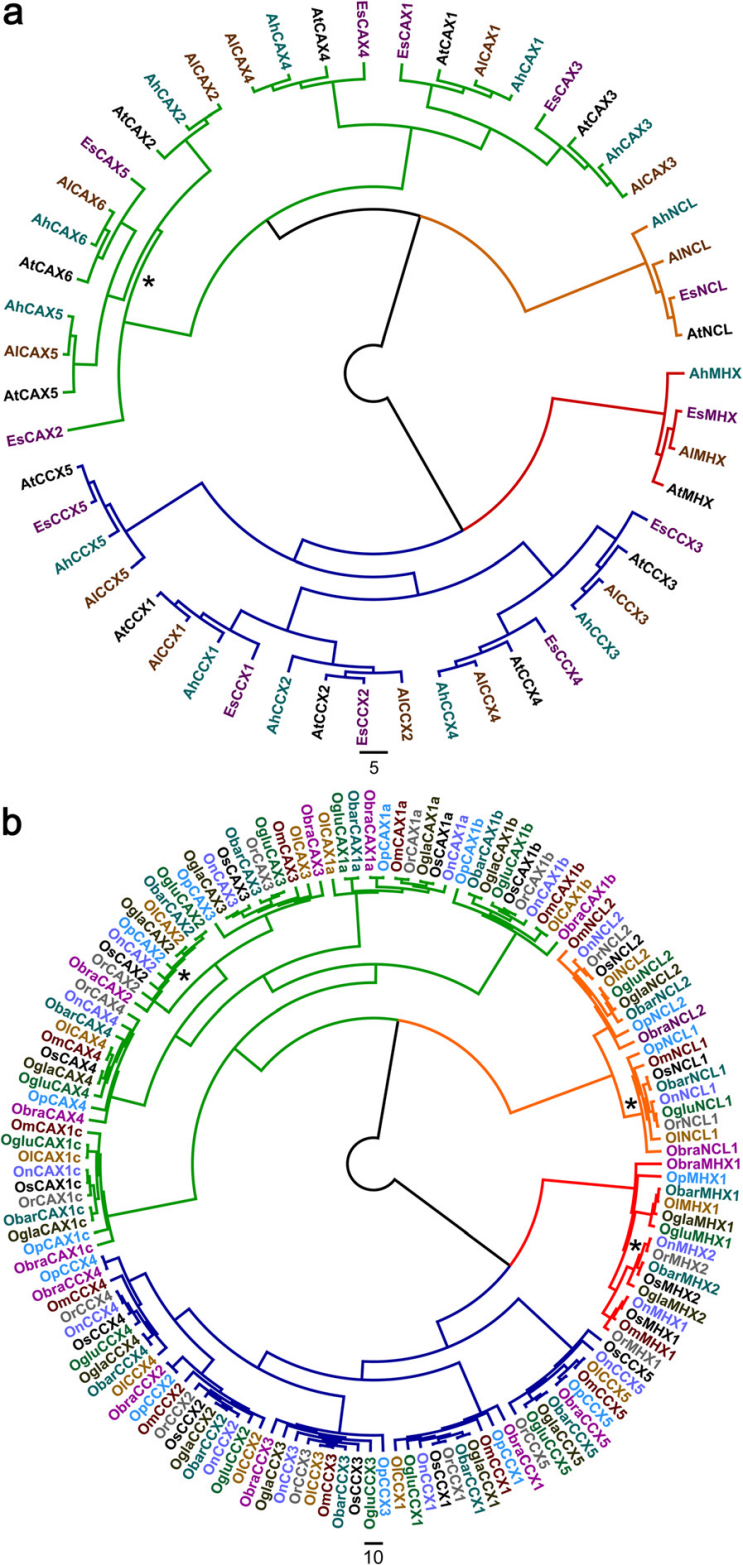
Conservation of CaCA genes in close relatives of *Arabidopsis thaliana* and *Oryza sativa*. **a** Phylogenetic analysis of CaCA proteins from *A. thaliana*, *A. halleri*, *A. lyrata* and *Eutrema salsugineum*. **b** Phylogenetic analysis of CaCA proteins from *O. sativa*, *Oryza barthii*, *O. brachyantha*, *O. glaberrima*, *O. glumipatula*, *O. longistaminata*, *O. meridionalis*, *O. nivara*, *O. punctate* and *O. rufipogon*. Evolutionary relationships of these rice species is shown in Additional file 5: Figure S3. CaCA gene families are colour coded as shown in Fig. 1. Phylogenetic analysis was performed as described in Fig. 1. Asterisks indicate gene clades where one or more species are absent

CaCA genes are ubiquitous within plants but can vary in number; CAX gene numbers vary from 4 in *Vitis vinifera* to 8 in *Glycine max* (Emery et al. 2012) while all plants have one MHX apart from *O. sativa* (Japonica and Indica) and *Mimulus guttatus*, which have two MHXs (Gaash et al. 2013). It is unknown whether CaCA gene family structures are conserved amongst closely related species. Here we surveyed the genomes of three species closely related to *A. thaliana*, and nine additional rice species, then phylogenetic analysis using full length sequences was performed. For Arabidopsis relatives each gene family is highly stable; each species has one MHX, one NCL, five CCXs and six CAXs, except that *E. salsugineum* has just two Type 1B CAXs (*CAX2*, *CAX5*, *CAX6*) (Fig. 2a). Gene family structure was also highly conserved within *Oryza* species: there were no differences between CCX genes, and all CAX and NCL genes were conserved, except that a *CAX2* gene was undetected in *O. meridionalis* and a *NCL1* gene was undetected in *O. glaberrima* (Fig. 2b). All rice genomes possessed a *MHX1* gene but *MHX2* was only found in five of the ten species. Gaash et al. (2013) suggested that MHX gene duplication occurred before the split between the *O. sativa* Indica and Japonica subspecies. Consistent with this conclusion, we found *MHX2* in four AA genome rice species (*O. barthii, O. glaberrima, O. nivara, O. rufipogon*), but not in the BB genome (*O. punctata*) or FF genome (*O. brachyantha*) species (Additional file 5: Figure S3), suggesting that gene duplication occurred during AA genome rice evolution but *MHX2* was subsequently lost in some AA genome species like *O. glumipatula* and *O. longistaminata*.

### Protein structure homology modelling supports CAX and NCX/MHX distinction

**Fig. 3:**
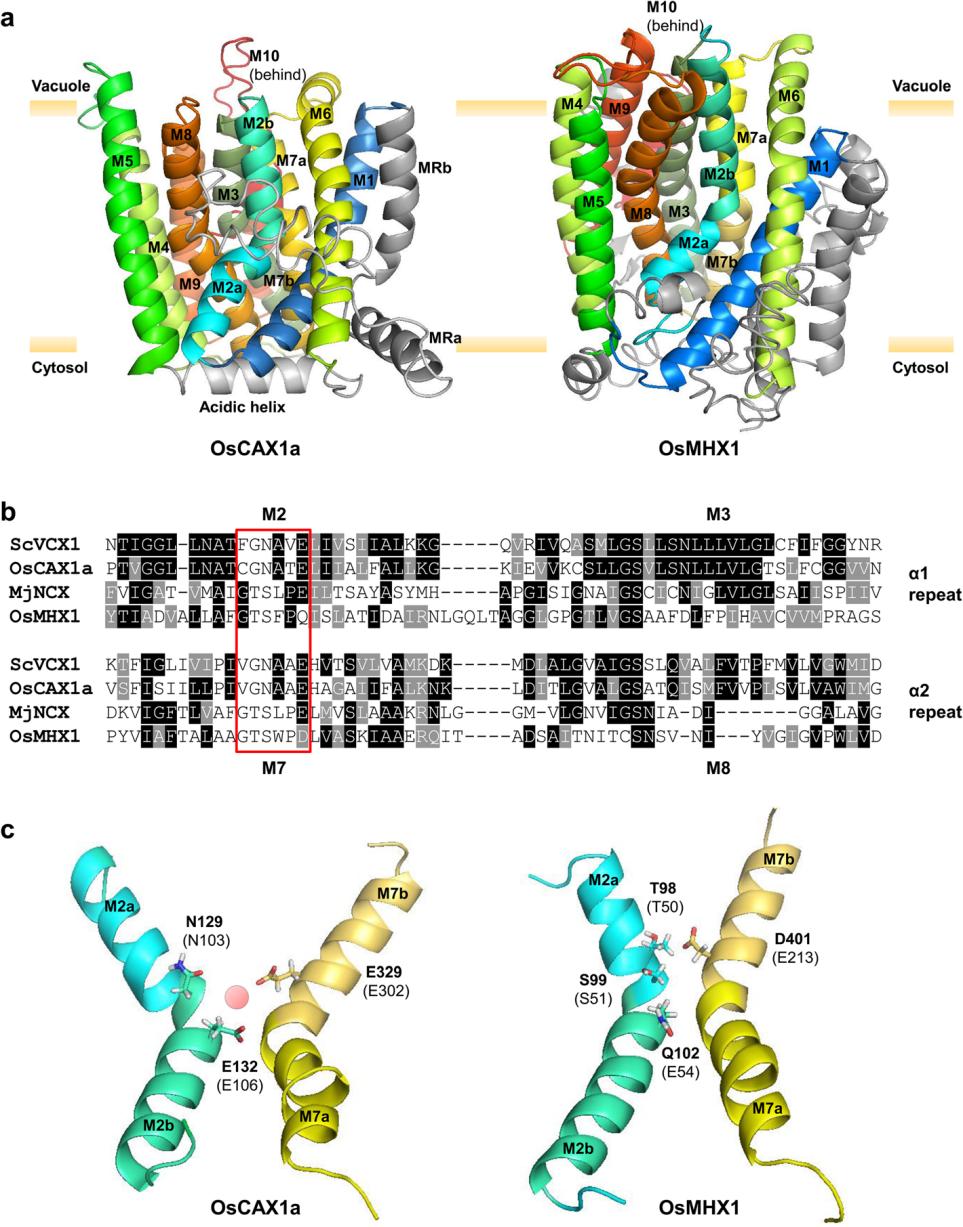
Comparative structure models of OsCAX1a and OsMHX1 determined by homology modelling. **a** Models of OsCAX1a and OsMHX1 generated using the known structures of ScVCX1 and MjNCX, respectively. The proteins are viewed from within the membrane. OsCAX1a has 11 predicted transmembrane (TM) helices with the first non-conserved TM labelled as MR. TM helices that can be clearly sub-divided (*kinked*) are referred to as ‘a’ and ‘b’. M1 to M10 in both proteins are coloured equivalently, and the MR and acidic helix domains, and non-TM regions are shaded in grey. **b** Sequence alignment of the α-repeat sequences within helices M2/M3 and M7/M8. The signature sequence (GNXXE for CAX; GXSXPE/D for NCX) is boxed in red. **c** Comparison of the putative cation binding pocket of OsCAX1a and OsMHX1. Key residues of M2 and M7 that bind to a Ca^2+^ ion in ScVCX1 (relative to OsCAX1a) and in MjNCX (relative to OsMHX1) are indicated in parentheses while the equivalent residues in the same positions are shown. A Ca^2+^ ion is indicated in the OsCAX1a pocket. Substitution of a Glu residue (E54) with a Gln residue (Q102) in OsMHX1 may explain the lack of Ca^2+^ binding but Mg^2+^-binding residues are unknown

Recent crystallographic analysis of MjNCX from *Methanococcus jannaschii* (Liao et al. 2012) and CAX proteins from archaea, bacteria and yeast (Nishizawa et al. 2013; Waight et al. 2013; Wu et al. 2013), including *Saccharomyces cerevisiae* ScVCX1, provided the first detailed structural information of CaCAs. Although MjNCX displays Na^+^/Ca^2+^ exchange activity, phylogenetically it is a YRBG member (Liao et al. 2012; Gaash et al. 2013), and related to MmYRBG1 (Fig. 1). However, MjNCX has previously been used to successfully generate protein homology models of NCX proteins from nematodes (He and O'Halloran 2014), and there is strong evidence that a 10 TM structure is conserved between NCX and MjNCX proteins (John et al. 2013; Ren and Philipson 2013). As yet, there is no structural information for plant exchangers. Using ScVCX1 and MjNCX structures as templates, protein homology modelling was performed for OsCAX1a and OsMHX1 (Additional file 1: Methods). A model of OsCAX1a was generated (Fig. 3a) showing high similarity to ScVCX1 (Additional file 6: Figure S4). OsMHX1 was harder to model, and despite showing some alignment with MjNCX, it was not identical, highlighting the distinction between a Na^+^/Ca^2+^ transporting YRBG family member and MHX proteins. Previous evaluation of MHX protein structure by comparison with NCX proteins and hydropathy prediction analysis suggested that MHX proteins have 9 TM helices (Gaash et al. 2013). However, this assumption was based on earlier biochemical models of a 9 TM topology for NCX1, but more recent studies demonstrate that NCX1 is composed of 10 TM helices (Ren and Philipson 2013; Szerencsei et al. 2013). This topology of NCX1 and consensus hydropathy prediction for MHX proteins (Additional file 7: Figure S5) provides confidence in this 10 TM helices model of OsMHX1. Despite overall conservation in core structure between CaCA proteins (Additional file 3: Figure S1a), there are clear structural differences between plant CAX and MHX (Fig. 3a), and non-plant CAX and NCX proteins (Additional file 8: Figure S6). For example, an additional redundant N-terminal TM helix (denoted MR) is found in most CAX proteins, and a cytosolic domain rich in acidic residues (the acidic helix) is a common feature linking the pseudo-symmetrical halves of CAX proteins (Fig. 3a). OsCAX1a also appears to be more tightly packed in the membrane than OsMHX1 (Additional file 3: Figure S1b).

Although the α1- and α2-repeat regions are highly conserved features of CaCAs, there is clear sequence variation, as demonstrated by the phylogenetic variation within the α2-repeat tree (Additional file 4: Figure S2c). Comparison of primary sequence for the α1- and α2-repeat regions from two CAX proteins and two NCX/MHX proteins demonstrates the sequence similarity within CaCA classes but sequence distinction between them (Fig. 3b). CAX proteins possess an α-repeat signature sequence of GNXXE while NCX proteins have a signature sequence of GXSXPE/D (Cai and Lytton 2004). The Glu (or Asp) residues within M2 and M7 are suggested to be critical in coordinating the geometry of the Ca^2+^ ion (Fig. 3c), with other residues acting to stabilise this coordination (Liao et al. 2012; Waight et al. 2013). These negatively charged Glu/Asp residues are highly conserved, with an exception being the non-charged Gln residue (Gln-102 in OsMHX1) within the MHX α1-repeat, suggested in part to explain the lack of Ca^2+^ binding by MHXs (Gaash et al. 2013). The M2 and M7 TM helices are strongly kinked, and this topology yields a cation binding pocket in combination with the tilted, weakly bent M3 and M8 helices (Additional file 3: Figure S1a) (Liao et al. 2012; Nishizawa et al. 2013). Nishizawa et al. (2013), Waight et al. (2013), and Wu et al. (2013) indicated clear differences in the kink angle of M2 between MjNCX and the CAX proteins (Additional file 8: Figure S6b). Furthermore, conformation of the M1/M6 ‘gating bundle’ is opposed in CAX relative to NCX (Additional file 8: Figure S6c). These structural variations explain the differences between the mutually-exclusive Ca^2+^ and H^+^ binding of CAX and the 1 Ca^2+^/3 Na^+^ binding of NCX (Nishizawa et al. 2013). MjNCX shows an outward-facing conformation while ScVXC1, AfCAX and YfkE have an inward-facing conformation (Nishizawa et al. 2013; Waight et al. 2013; Wu et al. 2013). OsCAX1a also displays inward facing conformation, while the closer similarity of OsMHX1 with CAX topologies compared to an NCX topology (Additional file 1: Methods), particularly with regard to the position of M6 (Additional file 6: Figure S4) suggests that MHX also shows an inward-facing conformation.

## Conclusions

NCX/MHX, NCKX, YRBG, CCX and CAX/NCL proteins are a unique family of ion exchangers that share conserved structural features but also clear distinctions. While phylogenetic distinctions between NCX and MHX genes or CAX and NCL genes could be argued, there is clear phylogenetic separation between the CAX/NCL, NCX/MHX, NCKX, YRBG and CCX families (Fig. 1). Thus it is certainly not supported for these proteins to be combined into a single gene family named ‘NCX’ as proposed by Singh et al. (2015); rather the evidence overwhelmingly shows that NCX is a separate family within the CaCA superfamily. Nomenclature changes can lead to confusion, but sometimes it is appropriate. The CaCA genes originally named *AtCAX7 – AtCAX11* were renamed *AtCCX1 – AtCCX5* after phylogenetic evidence clearly showed these genes to be members of the CCX rather than CAX family (Shigaki et al. 2006). However, it is not appropriate to change the CaCA gene nomenclature as proposed (Singh et al. 2015). More pertinently, there are clear functional differences between these proteins; for example, exchange of H^+^/Ca^2+^ (AtCAX1), H^+^/Mn^2+^ (AtCAX2), H^+^/Mg^2+^ (AtMHX), H^+^/K^+^ (AtCCX3), and Na^+^/Ca^2+^ (AtNCL) (Hirschi et al. 1996; Shaul et al. 1999; Pittman et al. 2004; Morris et al. 2008; Li et al. 2016), strongly argues against a common classification of these proteins as Na^+^/Ca^2+^ exchangers.

## Additional files

Additional file 1: Methods. Methods for CaCA sequence identification, phylogenetic analysis, and protein structure homology modelling. (PDF 165 kb) Additional file 2: Table S1. Reference number or accession number of the sequences used for the phylogenetic analysis. (XLSX 23 kb) Additional file 3: Figure S1. Predicted structure of a typical CaCA protein monomer based on experimentally determined and homology predicted structures of NCX and CAX proteins. Schematic representation of the topology of a typical CaCA protein monomer. Homology model structures of OsMHX1 (NCX/MHX family) and OsCAX1a (CAX family) from rice viewed above from the vacuolar lumen side. (PDF 240 kb) Additional file 4: Figure S2. Phylogenetic analysis of the CaCA superfamily. Trees derived from alignments of full-length sequences, conserved ‘core domain’ hydrophobic region sequences, and α2-repeat region sequences. (PNG 437 kb) Additional file 5: Figure S3. A schematic phylogenetic species tree showing the evolutionary relationship of the ten *Oryza* rice species compared in this study. (PDF 9 kb) Additional file 6: Figure S4. Structure alignments of OsCAX1a and OsMHX1 with ScVCX1 and MjNCX. (PDF 429 kb) Additional file 7: Figure S5. Hydropathy plot comparisons for AtCAX1, OsCAX1a, AtMHX1 and OsMHX1. (PDF 1385 kb) Additional file 8: Figure S6. Comparative structures of the yeast CAX protein ScVCX1 and the *Methanococcus jannaschii* NCX protein MjNCX. (PDF 313 kb)

## Abbreviations

CaCA: Ca^2+^/cation antiporter
CAX: H^+^/Cation exchanger
CCX: cation/Ca^2+^ exchanger
MHX: Mg^2+^/H^+^ exchanger
NCKX: Na^+^/Ca^2+^, K^+^ exchanger
NCL: Na^+^/Ca^2+^ exchanger-like
NCX: Na^+^/Ca^2+^ exchanger
TM: transmembrane
