# Supplementary material for: Phylogenetic analysis and protein structure modelling identifies distinct Ca2+/Cation antiporters and conservation of gene family structure within Arabidopsis and rice species
Source: Rice (N Y). 2016 Feb 1;9:3. doi: 10.1186/s12284-016-0075-8 (PMC4735048; doi:10.1186/s12284-016-0075-8)
Supplement: Additional file 1: Methods. — Methods for CaCA sequence identification, phylogenetic analysis, and protein structure homology modelling. (PDF 165 kb) [file 12284_2016_75_MOESM1_ESM.pdf]

## Additional file 1: Methods

### CaCA sequence identification

Sequences for comparative phylogenetic analysis were obtained from 13 species: two higher plants, *Arabidopsis thaliana* (Arabidopsis) and *Oryza sativa* Japonica (rice); one moss, *Physcomitrella patens*; three algae including a freshwater green microalga, *Chlamydomonas reinhardtii*, a marine diatom, *Phaeodactylum tricornutum*, and a marine coccolith, *Emiliania huxleyi*; one yeast, *Saccharomyces cerevisiae*; a protist, the human malarial parasite *Plasmodium falciparum*; two animals, the zebrafish *Danio rerio* and human *Homo sapiens*; the bacterium *Escherichia coli*; the cyanobacterium *Synechococcus* sp.; and the archaebacterium *Methanococcus maripaludis*. The sequences for all species were obtained from genome databases (listed in Additional file 2: Table S1) using BLAST as described by Emery et al. (2012). Rice and Arabidopsis genes from the CAX (cation/$H^+$ exchanger), CCX (cation/$Ca^{2+}$ exchanger), NCX/MHX ($Na^+/Ca^{2+}$ exchanger; $Mg^{2+}/H^+$ exchanger), and NCL ($Na^+/Ca^{2+}$ exchanger-like (Wang et al. 2012)) families were used, as described previously (Emery et al. 2012; Gaash et al. 2013; Singh et al. 2015), but with a few differences. In addition to the 6 CAX, 5 CCX and 1 MHX gene from Arabidopsis, only 1 NCL was included (*AtNCL*; At1g53210), while a second potential NCL gene (At1g29020, previously named AtEFCAX2 (Emery et al. 2012)) was discounted as further analysis of the putative α2-repeat region concluded that it lacked too many conserved residues that may be essential for $Ca^{2+}/Na^+$ exchange. From rice, 6 CAX and 2 NCL genes were included, as in previous analysis (Emery et al. 2012; Singh et al. 2015), plus two MHX genes as identified by Gaash et al. (2013). Emery et al. (2012) had identified four CCX genes from rice while Singh et al. (2015) identified two additional CCX genes, which were suggested to be ‘half’ proteins possessing single α-repeat regions. Manual annotation indicated that one of the genes (Os11g05070) had in fact been mis-annotated and a full length open reading frame (ORF) of the expected size and possessing both α-repeat regions could be determined (and was named *OsCCX5*). The second gene (Os11g01580) was very similar to *OsCCX3*, but a full length CCX ORF could not be identified, possibly due to poor genome sequence. However, Os11g01580 may either be a pseudogene resulting from a recent partial gene duplication event or a true ‘half-size’ CCX, but was not included in the phylogenetic analysis.

CaCA genes were identified from close relatives of Arabidopsis (*Arabidopsis halleri*, *Arabidopsis lyrata* and *Eutrema salsugineum* (formerly referred to as *Thellungiella halophila*)), and nine additional *Oryza* species: *Oryza barthii, Oryza brachyantha, Oryza glaberrima, Oryza glumipatula, Oryza longistaminata, Oryza meridionalis, Oryza nivara, Oryza punctate,* and *Oryza rufipogon*. Further rice species information is provided in Additional file 2: Table S1 and the evolutionary relationship between these rice species is

shown in Additional file 5: Figure S3. CaCA genes from Arabidopsis and rice relatives were identified by BLAST searches using CAX, CCX, MHX, and NCL genes from *A. thaliana* and *O. sativa*, respectively. *A. halleri*, *A. lyrata* and *E. salsugineum* genomes were searched using the genome browsers and BLAST on Phytozome (http://phytozome.jgi.doe.gov/; version 10.3; searched in August 2015), while the rice species genomes were searched using the genome browsers and BLAST on Gramene (http://www.gramene.org; version 47; searched in August 2015).

All sequences used for analysis are listed in Additional file 2: Table S1. For the visual analysis and comparison of CaCA protein sequences, multiple sequence alignments were performed using Clustal Omega (http://www.ebi.ac.uk/Tools/msa/clustalo/) and shaded using the BOXSHADE v.3.2 viewer and manually annotated where necessary.

**Transmembrane helix prediction**

Comparison of hydropathy prediction methods to determine transmembrane helix number and location within AtCAX1, OsCAX1a, AtMHX1 and OsMHX1 sequences was determined using 18 individual programs and three consensus programs available with the ARAMEMNON v.8.0 plant membrane protein database (Schwacke et al. 2003). The individual prediction programs used were Alom v.2, , DAS-TMfilter, Eiconda v.1, HmmTop v.2, MemSat v.3, Minnou, PHDhtm, Philius, Phobius, PredTmr v.1, Scampi, SosuiG v.1.1, SVMtm v.3, THUMBUP v.1, TmHMM v.2, TMMOD, TmPred and TopPred v.2, and three consensus prediction programs, AramTmCon, AramTmMultiCon and ConPred v.2.

**Phylogenetic analysis**

Analysis was performed using alignments of full-length amino acid sequences, of ‘core domain’ sequences, and of consensus α2-repeat region sequences extracted from each CaCA protein. The α2-repeat region sequences were identified from consensus sequence of previously determined CaCA proteins (Cai and Lytton 2004; Emery et al. 2012). Core hydrophobic domain sequences were extracted following removal of non-conserved sequence before and after the first and last conserved transmembrane domain for each protein sequence, and the large central loop region as determined by TMHMM v2.0 (www.cbc.dtu.dk/services/TMHMM). Sequences were aligned using MAFFT v6.0 (E-INS-i option). The phylogenies were estimated using maximum likelihood under the WAG+F model of amino acid substitution with Γ-distributed rates across sites, as implemented in RAxML v7.1, with tree confidence determined from 1000 replications using the fast bootstrap method (Stamatakis 2006; Stamatakis et al. 2008). The trees were viewed using FigTree v1.3 (http://tree.bio.ed.ac.uk/software/figtree). Trees for Arabidopsis relatives and rice relatives were only constructed using alignments of predominantly full length sequences,

although for a small number of the rice species genes, only partial length sequences were available (Additional file 2: Table S1).

**Protein structure homology modelling**

Protein structure prediction was performed for full length OsCAX1a and OsMHX1 amino acid sequences using the I-TASSER software server (Roy et al. 2010; Yang et al. 2015), using the 2.3 Å resolution crystal structure of the yeast vacuolar $Ca^{2+}/H^{+}$ exchanger ScVCX1 (Waight et al. 2013) (RCSB PDB id: 4k1cA), and the 1.9 Å resolution crystal structure of the *Methanococcus jannaschii* $Na^{+}/Ca^{2+}$ exchanger MjNCX (Liao et al. 2012) (RCSB PDB id: 3v5uA), respectively. Threading alignments were generated using the ten best PDB hits selected from multiple threading programs on the LOMETS meta-server. For OsCAX1a modelling, all ten hits were for the same protein structure: ScVCX1, with the highest normalised Z-score of 8.33 from the HHSEARCH program, demonstrating good alignment. The best OsCAX1a homology model had a confidence score (C-score) of -0.84, an estimated TM-score of 0.61 ± 0.14, and an estimated RMSD of 9.0 ± 4.6 Å. For OsMHX1 modelling, the best threading hits from three programs were for the MjNCX protein structure, with the highest normalised Z-score of 6.02 from the HHSEARCH program, also demonstrating good alignment. There were also four hits against the 2.3 Å resolution crystal structure of the *Archaeoglobus fulgidus* $Ca^{2+}/H^{+}$ exchanger protein AfCAX (Nishizawa et al. 2013) (RCSB PDB id: 4kppA), with the highest normalised Z-score of 2.37 from the Neff-PPAS program. The best OsMHX1 homology model had a C-score of -2.20, an estimated TM-score of 0.45 ± 0.15, and an estimated RMSD of 12.7 ± 4.2 Å. TM-align was used match the best homology models to all structures in the PDB library. The OsCAX1a model aligned strongly to ScVCX1 with a high TM-score of 0.82 (see Additional file 6: Figure S4). OsCAX1a also aligned strongly to AfCAX (TM-score 0.65) and to the 3.1 Å resolution crystal structure of the *Bacillus subtilis* $Ca^{2+}/H^{+}$ exchanger protein YfkE (Wu et al. 2013) (RCSB PDB id: 4kjsA) (TM-score 0.66). The OsMHX1 model aligned modestly to all four exchangers and with equivalent scores (AfCAX: TM-score 0.66; ScVCX1: TM-score 0.56; YfkE: TM-score 0.51; MjNCX: TM-score 0.47). All structural images were generated using PyMOL software.

**References**

Cai XJ, Lytton J (2004) The cation/$Ca^{2+}$ exchanger superfamily: Phylogenetic analysis and structural implications. Mol Biol Evol 21 (9):1692-1703

Emery L, Whelan S, Hirschi KD, Pittman JK (2012) Protein phylogenetic analysis of $Ca^{2+}$/cation antiporters and insights into their evolution in plants. Front Plant Sci 3:1. doi:10.3389/fpls.2012.00001

Gaash R, Elazar M, Mizrahi K, Avramov-Mor M, Berezin I, Shaul O (2013) Phylogeny and a structural model of plant MHX transporters. BMC Plant Biol 13 (1):75

Liao J, Li H, Zeng W, Sauer DB, Belmares R, Jiang Y (2012) Structural insight into the ion-exchange mechanism of the sodium/calcium exchanger. Science 335 (6069):686-690

Nishizawa T, Kita S, Maturana AD, Furuya N, Hirata K, Kasuya G, Ogasawara S, Dohmae N, Iwamoto T, Ishitani R, Nureki O (2013) Structural basis for the counter-transport mechanism of a $H^+/Ca^{2+}$ exchanger. Science 341 (6142):168-172

Roy A, Kucukural A, Zhang Y (2010) I-TASSER: a unified platform for automated protein structure and function prediction. Nat Protocols 5 (4):725-738

Schwacke R, Schneider A, van der Graaff E, Fischer K, Catoni E, Desimone M, Frommer WB, Flügge U-I, Kunze R (2003) ARAMEMNON, a novel database for Arabidopsis integral membrane proteins. Plant Physiol 131 (1):16-26

Singh A, Kumar R, Tripathi A, Gupta B, Pareek A, Singla-Pareek S (2015) Genome-wide investigation and expression analysis of Sodium/Calcium exchanger gene family in rice and Arabidopsis. Rice 8 (1):21

Stamatakis A (2006) RAxML-VI-HPC: Maximum likelihood-based phylogenetic analyses with thousands of taxa and mixed models. Bioinformatics 22 (21):2688-2690. doi:10.1093/bioinformatics/btl446

Stamatakis A, Hoover P, Rougemont J (2008) A rapid bootstrap algorithm for the RAxML web servers. Syst Biol 57 (5):758-771. doi:10.1080/10635150802429642

Waight AB, Pedersen BP, Schlessinger A, Bonomi M, Chau BH, Roe-Zurz Z, Risenmay AJ, Sali A, Stroud RM (2013) Structural basis for alternating access of a eukaryotic calcium/proton exchanger. Nature 499 (7456):107-110

Wang P, Li Z, Wei J, Zhao Z, Sun D, Cui S (2012) A $Na^+/Ca^{2+}$ exchanger-like protein (AtNCL) involved in salt stress in *Arabidopsis*. J Biol Chem 287 (53):44062-44070. doi:10.1074/jbc.M112.351643

Wu M, Tong S, Waltersperger S, Diederichs K, Wang M, Zheng L (2013) Crystal structure of $Ca^{2+}/H^+$ antiporter protein YfkE reveals the mechanisms of $Ca^{2+}$ efflux and its pH regulation. Proc Natl Acad Sci USA 110 (28):11367-11372. doi:10.1073/pnas.1302515110

Yang J, Yan R, Roy A, Xu D, Poisson J, Zhang Y (2015) The I-TASSER Suite: protein structure and function prediction. Nat Meth 12 (1):7-8
