# Supplementary material for: Phylogenetic analysis and protein structure modelling identifies distinct Ca2+/Cation antiporters and conservation of gene family structure within Arabidopsis and rice species
Source: Rice (N Y). 2016 Feb 1;9:3. doi: 10.1186/s12284-016-0075-8 (PMC4735048; doi:10.1186/s12284-016-0075-8)
Supplement: Additional file 3: Figure S1. — Predicted structure of a typical CaCA protein monomer based on experimentally determined and homology predicted structures of NCX and CAX proteins. Schematic representation of the topology of a typical CaCA protein monomer. Homology model structures of OsMHX1 (NCX/MHX family) and OsCAX1a (CAX family) from rice viewed above from the vacuolar lumen side. (PDF 240 kb) [file 12284_2016_75_MOESM3_ESM.pdf]

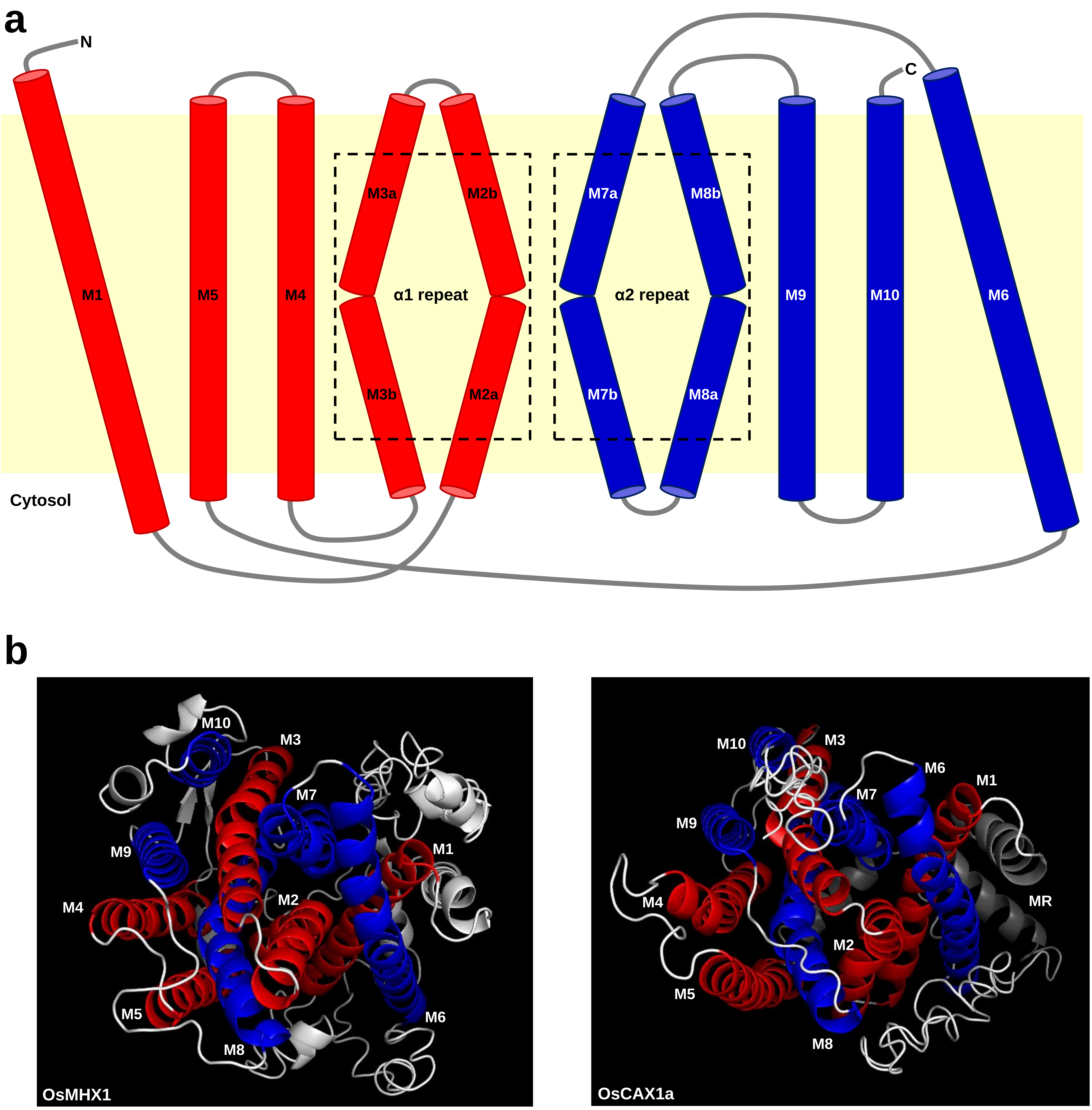

Additional file 3: Figure S1. Predicted structure of a typical CaCA protein monomer based on experimentally determined and homology predicted structures of NCX and CAX proteins. **a** Schematic representation of the topology of a typical CaCA protein monomer. The pseudo-symmetry of the N- and C-terminal halves of the protein is indicated by the red and blue coloured transmembrane (TM) spanning helices (M1-M5, M6-M10), respectively. The longer M1 and M6 helices form the proposed 'gating bundle'. The conserved α-repeat region sequences, which form the cation binding pocket, are indicated. Non-conserved features including additional 'redundant' TM helices (such as MR) and NCX family- and CAX family-specific domains (such as the acidic helix) on the large intracellular loop are not shown. **b** Homology model structures of OsMHX1 (NCX/MHX family) and OsCAX1a (CAX family) from rice viewed above from the vacuolar lumen side. N- and C-terminal half TM domains are coloured as shown in the topology model. The MR TM and acidic motif in the OsCAX1a model are shown in grey.
