# Supplementary figures and images for: Phylogenetic analysis and protein structure modelling identifies distinct Ca2+/Cation antiporters and conservation of gene family structure within Arabidopsis and rice species
Source: Rice (N Y). 2016 Feb 1;9:3. doi: 10.1186/s12284-016-0075-8 (PMC4735048; doi:10.1186/s12284-016-0075-8)

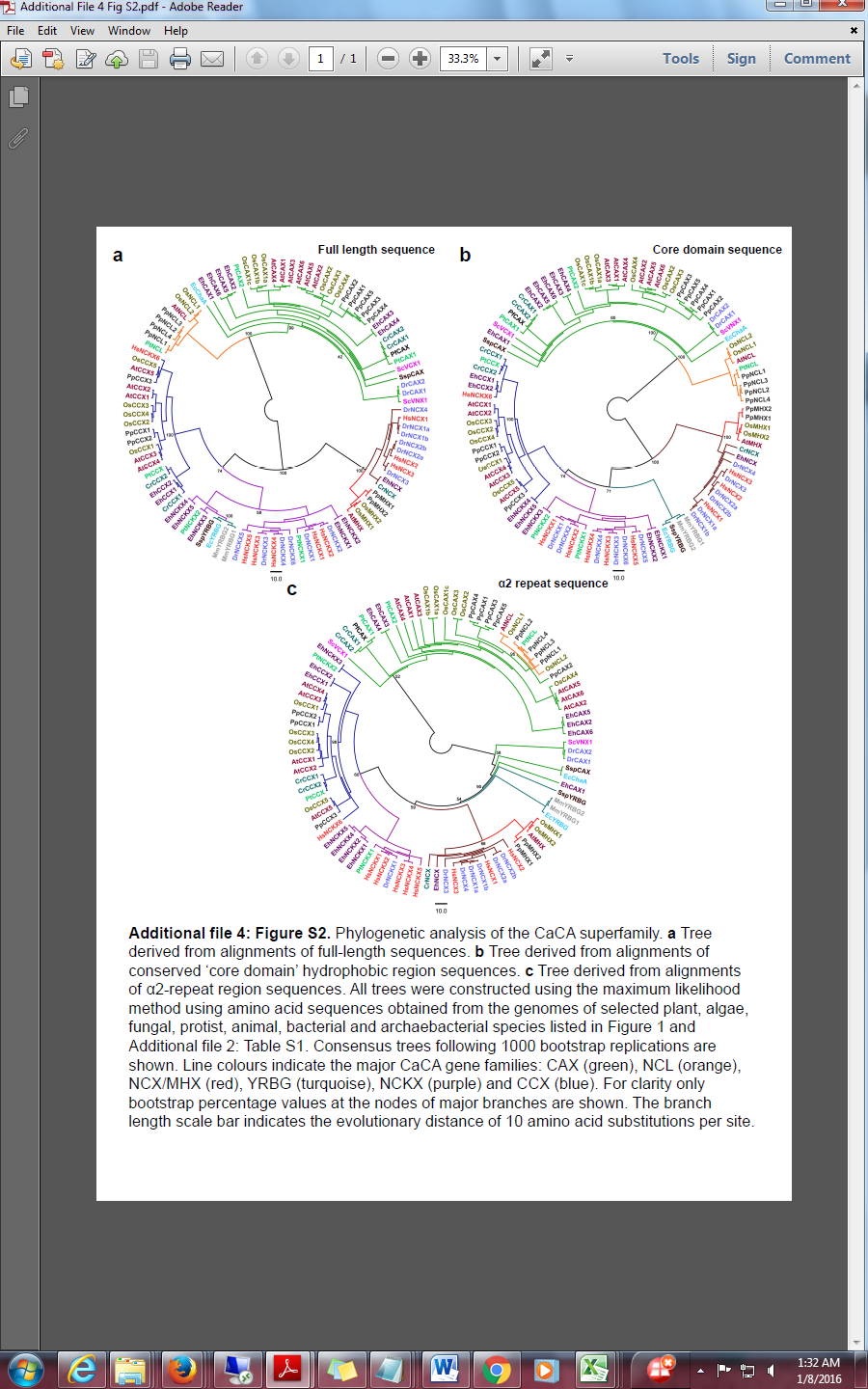

Supplement: Additional file 4: Figure S2. — Phylogenetic analysis of the CaCA superfamily. Trees derived from alignments of full-length sequences, conserved ‘core domain’ hydrophobic region sequences, and α2-repeat region sequences. (PNG 437 kb) [file 12284_2016_75_MOESM4_ESM.png]
