# Supplementary material for: Phylogenetic analysis and protein structure modelling identifies distinct Ca2+/Cation antiporters and conservation of gene family structure within Arabidopsis and rice species
Source: Rice (N Y). 2016 Feb 1;9:3. doi: 10.1186/s12284-016-0075-8 (PMC4735048; doi:10.1186/s12284-016-0075-8)
Supplement: Additional file 5: Figure S3. — A schematic phylogenetic species tree showing the evolutionary relationship of the ten Oryza rice species compared in this study. (PDF 9 kb) [file 12284_2016_75_MOESM5_ESM.pdf]

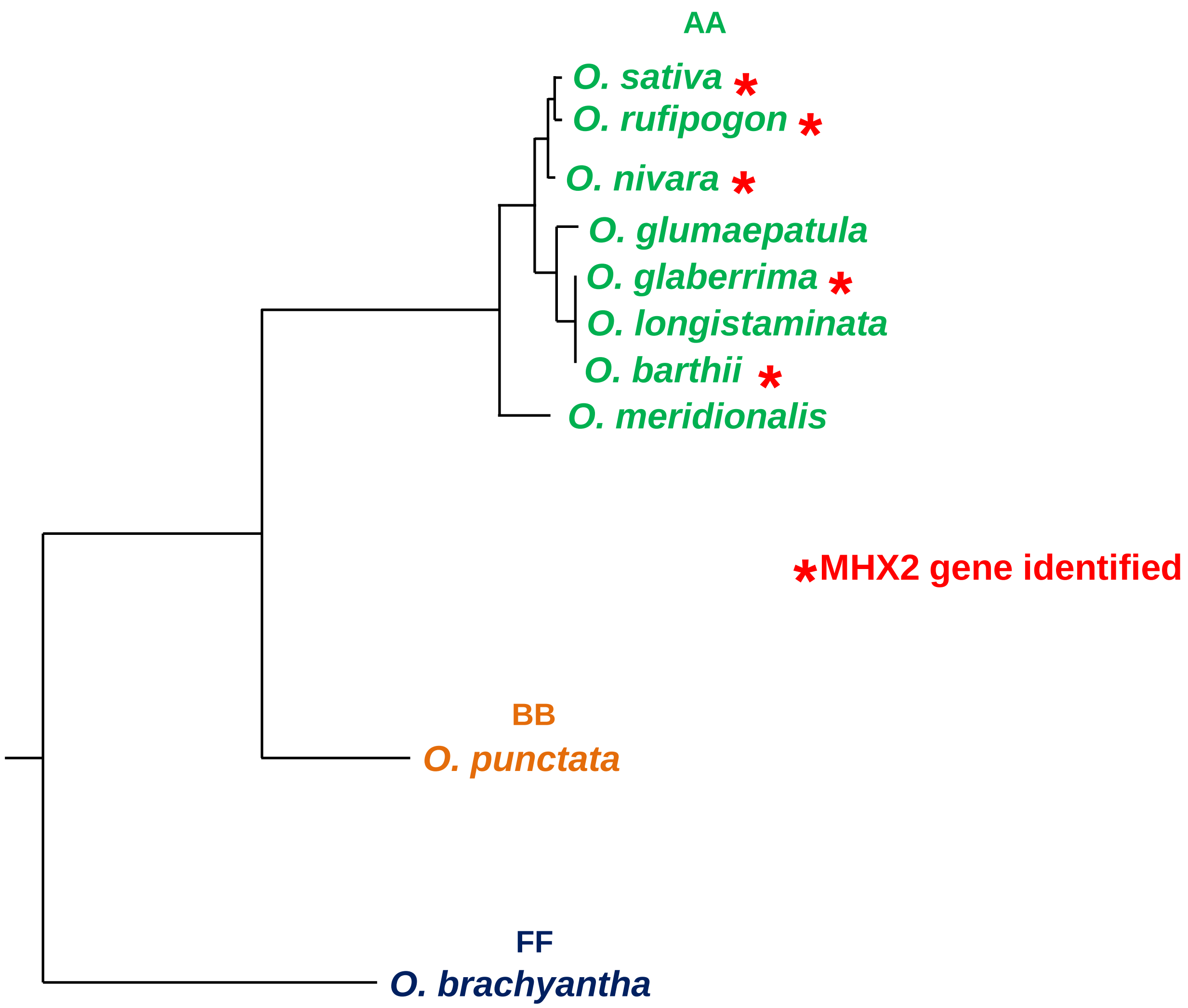

Additional file 5: Figure S3. A schematic phylogenetic species tree showing the evolutionary relationship of the ten *Oryza* rice species compared in this study. The tree is based on *Adh2* gene phylogeny performed by Ge et al. (1999)[1]. The eight AA genome species are shown in green, the BB genome species in orange, and the FF genome species in blue. The species that possess an *MHX2* gene, as analysed in Fig. 2, are indicated by an asterisk.

[1]Ge S, Sang T, Lu BR, Hong DY (1999) Phylogeny of rice genomes with emphasis on origins of allotetraploid species. Proc Natl Acad Sci U S A 96:14400-14405
