# Supplementary material for: Phylogenetic analysis and protein structure modelling identifies distinct Ca2+/Cation antiporters and conservation of gene family structure within Arabidopsis and rice species
Source: Rice (N Y). 2016 Feb 1;9:3. doi: 10.1186/s12284-016-0075-8 (PMC4735048; doi:10.1186/s12284-016-0075-8)
Supplement: Additional file 6: Figure S4. — Structure alignments of OsCAX1a and OsMHX1 with ScVCX1 and MjNCX. (PDF 429 kb) [file 12284_2016_75_MOESM6_ESM.pdf]

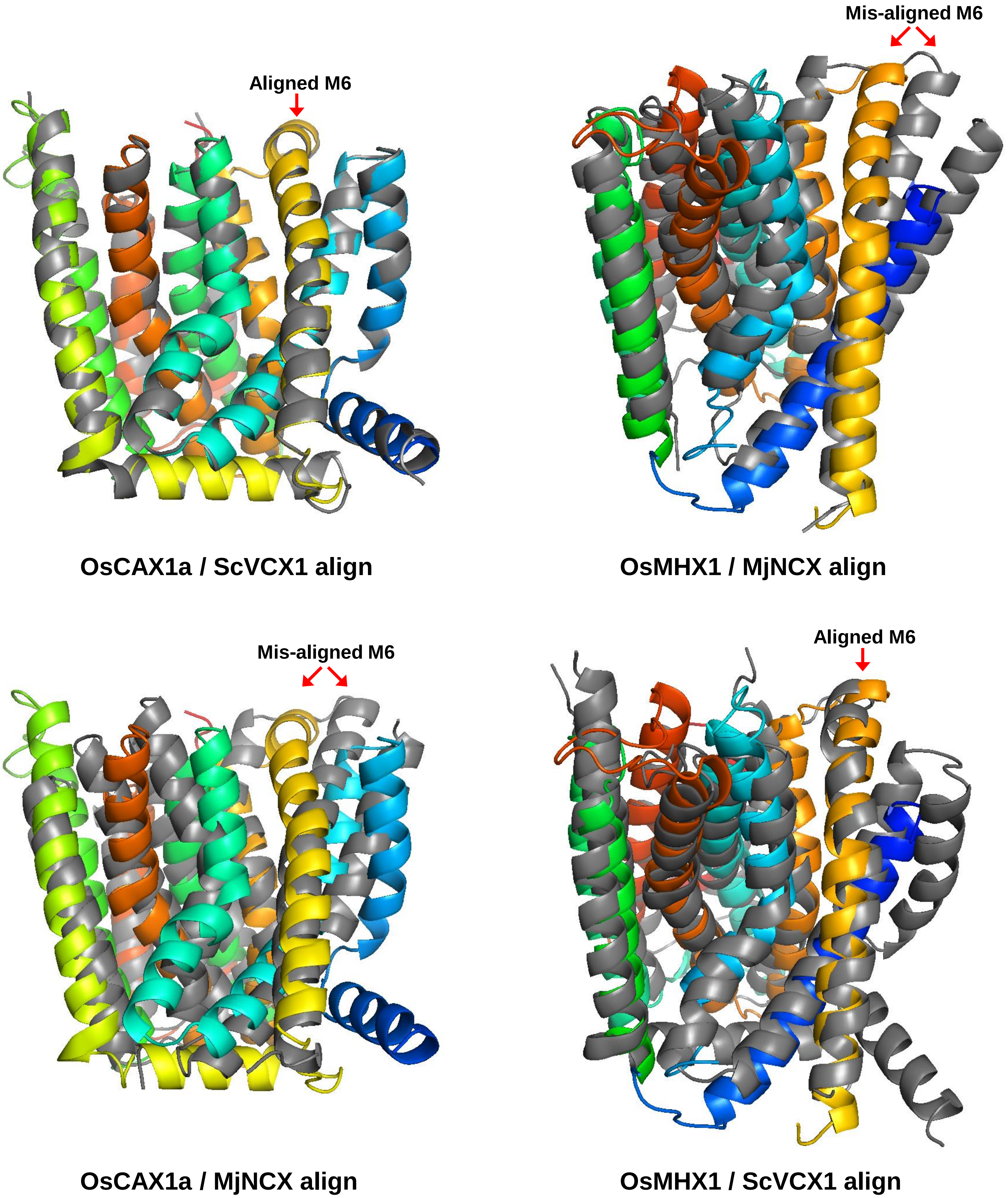

Additional file 6: Figure S4. Structure alignments of OsCAX1a and OsMHX1 with ScVCX1 and MjNCX. OsCAX1a and OsMHX1 structure models are shown in colour, ScVCX1 and MjNCX structures are shown in grey. OsCAX1a aligns very strongly with ScVCX1 but weakly with MjNCX, while OsMHX1 aligns weakly with MjNCX and with ScVCX1. The alignment of M6 helices are indicated. To improve the clarity of alignments, the lumenal loop region and hydrophilic terminal tail regions of OsMHX1 were removed.
