# Supplementary material for: Phylogenetic analysis and protein structure modelling identifies distinct Ca2+/Cation antiporters and conservation of gene family structure within Arabidopsis and rice species
Source: Rice (N Y). 2016 Feb 1;9:3. doi: 10.1186/s12284-016-0075-8 (PMC4735048; doi:10.1186/s12284-016-0075-8)
Supplement: Additional file 7: Figure S5. — Hydropathy plot comparisons for AtCAX1, OsCAX1a, AtMHX1 and OsMHX1. (PDF 1385 kb) [file 12284_2016_75_MOESM7_ESM.pdf]

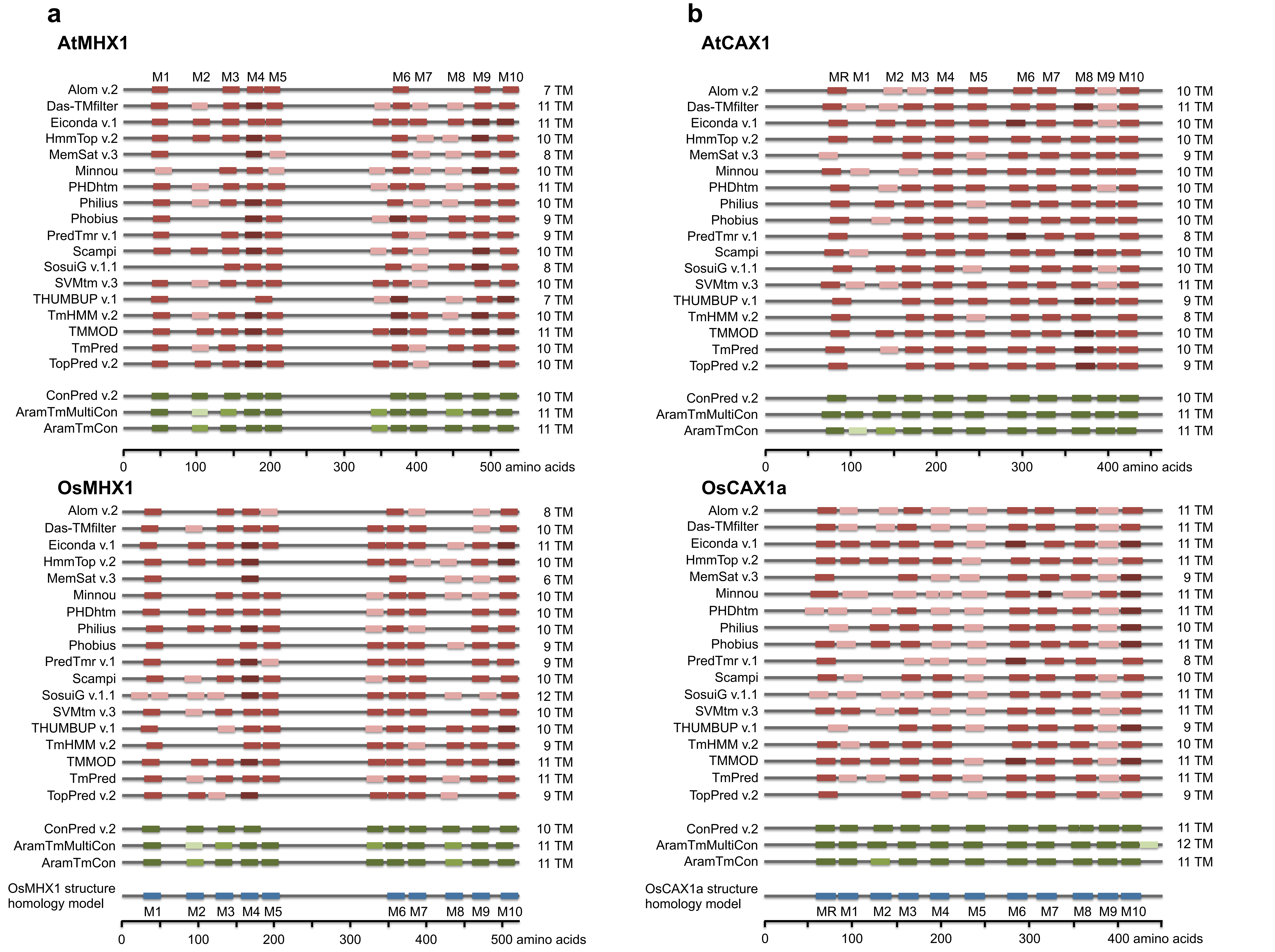

Additional file 7: Figure S5. Hydropathy plot comparisons for AtCAX1, OsCAX1a, AtMHX1 and OsMHX1. Schematic representation of the predicted topology of AtMHX1 and OsMHX1 (**a**), and AtCAX1 and OsCAX1a (**b**) as determined by 18 individual hydropathy prediction programs (results plotted in red) and 3 consensus prediction programs (results plotted in green) using the ARAMEMNON v.8 plant membrane protein database. The relative confidence scores from the hydropathy predictions are indicated by light (low score) to dark (high score) shading. The topology of OsMHX1 and OsCAX1a as determined by the structure homology models are plotted in blue. The predicted transmembrane (TM) helices are indicated as M1 to M10, with the redundant first CAX TM helix indicated as MR. Most of the programs weakly predict TM helices M2 and M8 in the MHX1 proteins and frequently predict a TM helix before M6, and the consensus programs suggest topologies of 10 or 11 TM helices for AtMHX1 and OsMHX1, in agreement with the OsMHX1 homology model. Prediction of TM helices are more consistent for the CAX proteins, with TM helices M1 and M2 most weakly predicted, particularly for AtCAX1, with the consensus programs almost consistently suggesting a topology of 11 TM helices for AtCAX1 and OsCAX1a, in strong agreement with the OsCAX1a homology model.
