# Supplementary material for: Phylogenetic analysis and protein structure modelling identifies distinct Ca2+/Cation antiporters and conservation of gene family structure within Arabidopsis and rice species
Source: Rice (N Y). 2016 Feb 1;9:3. doi: 10.1186/s12284-016-0075-8 (PMC4735048; doi:10.1186/s12284-016-0075-8)
Supplement: Additional file 8: Figure S6. — Comparative structures of the yeast CAX protein ScVCX1 and the Methanococcus jannaschii NCX protein MjNCX. (PDF 313 kb) [file 12284_2016_75_MOESM8_ESM.pdf]

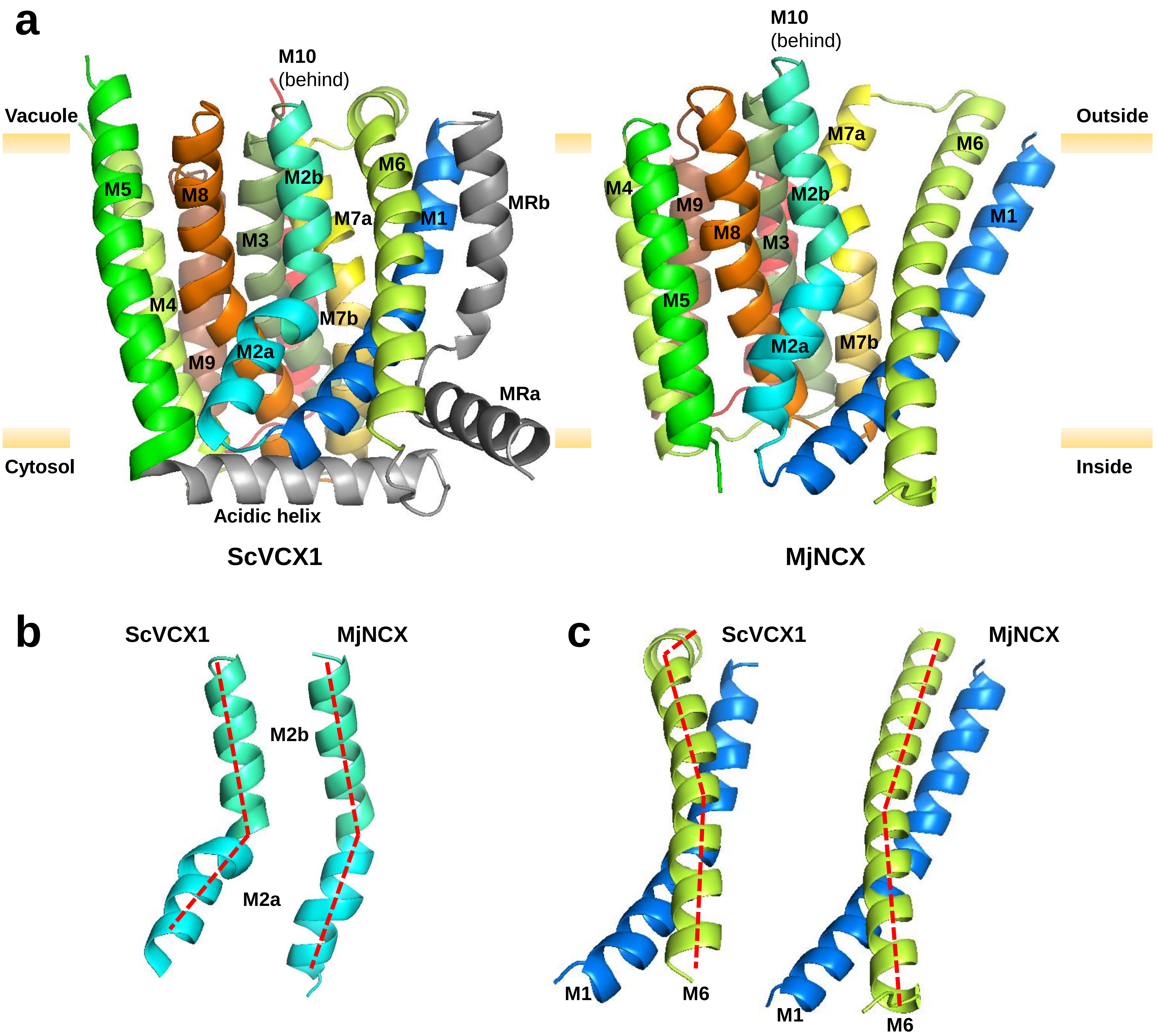

Additional file 8: Figure S6. Comparative structures of the yeast CAX protein ScVCX1 and the *Methanococcus* NCX protein MjNCX. **a** Structural characteristics of ScVCX1 and MjNCX by Waight et al. (2013)[1] and Liao et al. (2012)[2], respectively, were used to generate homology models of OsCAX1a and OsMHX1, respectively, shown in Fig. 3. The proteins are viewed from within the membrane. ScVCX1 has 11 transmembrane (TM) helices with the first non-conserved TM labelled as MR. TM helices that can be clearly sub-divided (kinked) are referred to as 'a' and 'b'. M1 to M10 in both proteins are coloured equivalently, and the MR and acidic helices in ScVCX1 are in grey. For MjNCX the disordered intracellular loop region between M5 and M6 is not shown. **b** Comparison of M2 helices with the altered kink angle indicated by the dashed lines. **c** Comparison of the M1/M6 'gating bundle' helices with the opposite orientation of M6 indicated by the dashed line.

[1]Waight AB, Pedersen BP, Schlessinger A, Bonomi M, Chau BH, Roe-Zurz Z, Risenmay AJ, Sali A, Stroud RM (2013) Structural basis for alternating access of a eukaryotic calcium/proton exchanger. Nature 499:107-110

[2]Liao J, Li H, Zeng W, Sauer DB, Belmares R, Jiang Y (2012) Structural insight into the ion-exchange mechanism of the sodium/calcium exchanger. Science 335:686-690
